# Supplementary material for: Involuntary temporary work and mental health medications: A longitudinal study in Denmark
Source: PLOS Glob Public Health. 2023 Nov 30;3(11):e0002634. doi: 10.1371/journal.pgph.0002634 (PMC10688703; doi:10.1371/journal.pgph.0002634)
Supplement: S1 Table — (DOCX) [file pgph.0002634.s001.docx]

**S1 Table.** Involuntary temporary full-time employment and mental health for men in different age classes, fixed effects estimation, quarterly observations, 2006-2018. Dependent variable indicator for drug prescription each quarter.

|  | Age Groups | | | |
| --- | --- | --- | --- | --- |
|  | (1) | (2) | (3) | (4) |
|  | 20-29 | 30-39 | 40-49 | 50-64 |
| Pre-treatment, 1 quarter | 0.0020 | 0.0073 | 0.0066 | -0.0131 |
|  | (0.0047) | (0.0073) | (0.0111) | (0.0097) |
| Temporary employment |  |  |  |  |
| Quarter 1 | 0.0048 | 0.0070 | -0.0024 | -0.0090 |
|  | (0.0065) | (0.0083) | (0.0158) | (0.0107) |
| Quarter 1-2 | -0.0123 | 0.0158 | -0.0246 | -0.0329** |
|  | (0.0104) | (0.0121) | (0.0204) | (0.0154) |
| Quarter 1-4 | 0.0168 | -0.0210 | 0.0714 | -0.0257 |
|  | (0.0197) | (0.0163) | (0.0642) | (0.0627) |
| Quarter 1-5 | -0.0014 | 0.0262 | -0.0066 | 0.0510 |
|  | (0.0059) | (0.0243) | (0.0296) | (0.0375) |
| Quarter 1-6 | 0.0000 | 0.0162 | -0.0159 | -0.0321* |
|  | (.) | (0.0168) | (0.0148) | (0.0193) |
| Post treatment | 0.0053 | 0.0097 | 0.0168 | -0.0102 |
|  | (0.0067) | (0.0104) | (0.0164) | (0.0114) |
| Post treatment, quarter 1-6 | 0.0000 | 0.0986 | -0.0385* | -0.4859** |
|  | (.) | (0.0832) | (0.0200) | (0.2309) |
| Education level |  |  |  |  |
| Low | 0.0115 | 0.1123 | 0.0262 | -0.4857 |
|  | (0.0183) | (0.0864) | (0.3211) | (0.3706) |
| High | 0.0019 | 0.0461 | 0.1958 | 0.0128 |
|  | (0.0061) | (0.0620) | (0.1926) | (0.0297) |
| White collar | -0.0034 | -0.0120 | 0.0141 | 0.0034 |
|  | (0.0079) | (0.0187) | (0.0190) | (0.0178) |
| Married | -0.0034 | 0.0023 | 0.0021 | 0.0202 |
|  | (0.0082) | (0.0126) | (0.0458) | (0.0489) |
| Children up to 6 years old | -0.0241 | -0.0195 | 0.0523 | 0.0688** |
|  | (0.0228) | (0.0210) | (0.0339) | (0.0328) |
| Children up to 18 years old | -0.0122 | 0.0516* | -0.0308 | -0.0809** |
|  | (0.0104) | (0.0297) | (0.0302) | (0.0322) |
| Income, normalized | -0.0027 | -0.0008 | -0.0206* | -0.0037 |
|  | (0.0050) | (0.0075) | (0.0107) | (0.0064) |
| Constant | 0.0328*** | 0.0077 | 0.0712 | 0.4436*** |
|  | (0.0102) | (0.0327) | (0.1172) | (0.0969) |
| No. of workers | 1,215 | 850 | 690 | 1,072 |

***Notes***: Significance levels: * 10%, ** 5%, *** 1%. Standard errors in parentheses are clustered at the person level and calculated using sample weights. Age is assessed at the time of the first interview. The indicator for temporary employment for, e.g., Quarter 1-6 takes the value 1 for each of the 6 quarters of temporary employment and 0 otherwise. The reference person is a blue-collar worker with middle-level education in the first quarter of 2012. The data encompasses persons who had full-time employment in at least one of the LFS surveys in 2006-2018 and include mental health indicators of the participants eight quarters before the first survey and eight quarters after the last survey.
