# Supplementary material for: Involuntary temporary work and mental health medications: A longitudinal study in Denmark
Source: PLOS Glob Public Health. 2023 Nov 30;3(11):e0002634. doi: 10.1371/journal.pgph.0002634 (PMC10688703; doi:10.1371/journal.pgph.0002634)
Supplement: S2 Table — (DOCX) [file pgph.0002634.s002.docx]

**S2 Table.** Women in involuntary temporary full-time employment and mental health according to labour market state before temporary employment, quarterly observations, 2006-2018. Dependent variable: indicator for drug prescription each quarter.

|  | OLS estimation | | | |  | Fixed effect estimation | | | |
| --- | --- | --- | --- | --- | --- | --- | --- | --- | --- |
|  | Full-time | Part-time  permanent | Part-time  temporary | Unemployed |  | Full-time | Part-time  permanent | Part-time  temporary | Unemployed |
|  | (1) | (2) | (3) | (4) |  | (5) | (6) | (7) | (8) |
| Pre-treatment, 1 quarter | -0.0034 | -0.0191 | -0.0281* | 0.0028 |  | -0.0013 | -0.0064 | -0.0168** | -0.0123 |
|  | (0.0076) | (0.0233) | (0.0163) | (0.0098) |  | (0.0087) | (0.0224) | (0.0083) | (0.0122) |
| Temporary employment | |  |  |  |  |  |  |  |  |
| Quarter 1 | -0.0070 | -0.0389 | -0.0020 | 0.0176 |  | -0.0067 | -0.0216 | -0.0063 | -0.0248 |
|  | (0.0110) | (0.0300) | (0.0193) | (0.0160) |  | (0.0110) | (0.0300) | (0.0103) | (0.0169) |
| Quarter 1-2 | 0.0503 | 0.0027 | -0.0748** | 0.0816 |  | 0.0237 | 0.0128 | 0.0099 | -0.0157 |
|  | (0.0428) | (0.0785) | (0.0376) | (0.0520) |  | (0.0305) | (0.0265) | (0.0276) | (0.0233) |
| Quarter 1-4 | -0.0671 | -0.0077 | -0.0726 | -0.0687 |  | -0.0179 | 0.0000 | -0.0050 | -0.0114 |
|  | (0.0549) | (0.1344) | (0.0642) | (0.0479) |  | (0.0156) | (0.0313) | (0.0114) | (0.0352) |
| Quarter 1-5 | 0.0143 | -0.0682 | -0.0315 | -0.0654 |  | 0.1276 | 0.0005 | 0.0101 | 0.0434 |
|  | (0.0747) | (0.0633) | (0.0414) | (0.0431) |  | (0.1058) | (0.0380) | (0.0106) | (0.0284) |
| Post treatment | -0.0077 | -0.0312 | -0.0342 | 0.0416** |  | -0.0127 | 0.0112 | -0.0013 | -0.0190 |
|  | (0.0117) | (0.0221) | (0.0240) | (0.0197) |  | (0.0135) | (0.0375) | (0.0121) | (0.0186) |
| Education level | |  |  |  |  |  |  |  |  |
| Low | -0.0412 | 0.1225 | 0.0964 | -0.0064 |  | -0.0447 | -0.0872 | 0.0067 | -0.0332 |
|  | (0.0348) | (0.0748) | (0.0842) | (0.0438) |  | (0.1041) | (0.0953) | (0.0648) | (0.0389) |
| High | -0.0083 | -0.1255** | -0.0266 | 0.0214 |  | 0.0087 | -0.0180 | -0.0392 | -0.0791** |
|  | (0.0284) | (0.0601) | (0.0506) | (0.0313) |  | (0.0546) | (0.0363) | (0.0274) | (0.0360) |
| White collar | -0.0473** | 0.0948* | -0.1084*** | -0.0541** |  | -0.0025 | 0.0452** | 0.0142 | 0.0113 |
|  | (0.0240) | (0.0503) | (0.0399) | (0.0251) |  | (0.0167) | (0.0211) | (0.0146) | (0.0176) |
| Married | -0.0419* | -0.0523 | -0.0429 | -0.0291 |  | 0.0058 | -0.0329 | -0.0090 | 0.0001 |
|  | (0.0235) | (0.0496) | (0.0422) | (0.0306) |  | (0.0211) | (0.0386) | (0.0215) | (0.0271) |
| Children up to 6 years old | -0.0626 | -0.1062 | 0.1441 | 0.0640 |  | -0.0091 | -0.1370** | 0.0328 | -0.1139 |
|  | (0.0381) | (0.0643) | (0.1069) | (0.0585) |  | (0.0352) | (0.0572) | (0.0390) | (0.0796) |
| Children up to 18 years old | -0.0112 | -0.0362 | 0.0562 | -0.0713* |  | -0.0369 | 0.0135 | 0.0354** | -0.0171 |
|  | (0.0317) | (0.0452) | (0.0608) | (0.0425) |  | (0.0246) | (0.0341) | (0.0175) | (0.0375) |
| Income, normalized | -0.0033 | -0.0445 | -0.0163 | 0.0081 |  | 0.0033 | 0.0126 | 0.0023 | -0.0008 |
|  | (0.0094) | (0.0280) | (0.0238) | (0.0165) |  | (0.0052) | (0.0154) | (0.0071) | (0.0156) |
| Constant | 0.2052*** | 0.2073*** | 0.2017*** | 0.2102*** |  | 0.2175*** | 0.1070 | 0.1363*** | 0.2995*** |
|  | (0.0411) | (0.0760) | (0.0621) | (0.0566) |  | (0.0538) | (0.0950) | (0.0375) | (0.0574) |
| No. of workers | 845 | 211 | 246 | 680 |  | 845 | 211 | 246 | 680 |

***Notes***: Significance levels: * 10%, ** 5%, *** 1%. Standard errors in parentheses are clustered at the person level and calculated using sample weights. Samples: Treatment groups where the labour market state before entering temporary full-time employment is available from the survey. Original labour market state: (1) Full-time permanent employment, (2) part-time permanent employment, (3) part-time temporary employment, and (4) unemployment. All regressions contain indicator variable controls for age and quarter. Pre-treatment is an indicator variable for the last quarter before entering the first treatment. The indicator for temporary employment for, e.g., Quarter 1-5 takes the value 1 for each of the 5 quarters of temporary employment and 0 otherwise. The reference person is a blue-collar worker with middle-level education of age 35-39 in the first quarter of 2012.
