# Supplementary material for: Involuntary temporary work and mental health medications: A longitudinal study in Denmark
Source: PLOS Glob Public Health. 2023 Nov 30;3(11):e0002634. doi: 10.1371/journal.pgph.0002634 (PMC10688703; doi:10.1371/journal.pgph.0002634)
Supplement: S3 Table — (DOCX) [file pgph.0002634.s003.docx]

**S3 Table.** Men in involuntary temporary full-time employment and mental health according to labour market state before temporary employment, quarterly observations, 2006-2018. Dependent variable: indicator for drug prescription each quarter.

|  | OLS estimation | | | |  | Fixed effect estimation | | | |
| --- | --- | --- | --- | --- | --- | --- | --- | --- | --- |
|  | Full-time | Part-time  permanent | Part-time  temporary | Unemployed |  | Full-time | Part-time  permanent | Part-time  temporary | Unemployed |
|  | (1) | (2) | (3) | (4) |  | (5) | (6) | (7) | (8) |
| Pre-treatment, 1 quarter | 0.0129 | -0.0102 | 0.0185 | -0.0131 |  | 0.0055 | -0.0254 | -0.0005 | -0.0095 |
|  | (0.0085) | (0.0307) | (0.0245) | (0.0128) |  | (0.0065) | (0.0274) | (0.0227) | (0.0112) |
| Temporary employment |  |  |  |  |  |  |  |  |  |
| Quarter 1 | 0.0051 | 0.0089 | 0.0029 | -0.0161 |  | 0.0038 | 0.0017 | -0.0265 | -0.0247* |
|  | (0.0105) | (0.0256) | (0.0261) | (0.0161) |  | (0.0084) | (0.0203) | (0.0210) | (0.0130) |
| Quarter 1-2 | 0.0623 | -0.0177 | -0.0836 | -0.0706* |  | -0.0119 | -0.0401 | 0.0528 | -0.0347** |
|  | (0.0461) | (0.0724) | (0.0670) | (0.0398) |  | (0.0168) | (0.0360) | (0.0483) | (0.0162) |
| Quarter 1-4 | 0.0743 | 0.0091 | 0.0966 | -0.0029 |  | 0.0518 | -0.0411 | -0.0420 | -0.0071 |
|  | (0.0904) | (0.0349) | (0.2820) | (0.0908) |  | (0.0541) | (0.0363) | (0.0587) | (0.0248) |
| Quarter 1-5 | 0.2338* | -0.4918*** | 0.0000 | -0.0837* |  | 0.0870* | -0.0713* | 0.0000 | -0.0131 |
|  | (0.1220) | (0.1331) | (.) | (0.0436) |  | (0.0486) | (0.0403) | (.) | (0.0175) |
| Post treatment | 0.0229* | 0.0250 | -0.0105 | -0.0138 |  | 0.0103 | 0.0057 | -0.0481 | -0.0135 |
|  | (0.0125) | (0.0290) | (0.0359) | (0.0163) |  | (0.0096) | (0.0212) | (0.0461) | (0.0146) |
| Education level |  |  |  |  |  |  |  |  |  |
| Low | -0.0112 | -0.0178 | 0.1715* | 0.0311 |  | 0.0088 | -0.2281 | 0.0348 | 0.0690 |
|  | (0.0272) | (0.0464) | (0.0871) | (0.0402) |  | (0.0170) | (0.1903) | (0.0550) | (0.0494) |
| High | -0.0027 | -0.0185 | -0.0525 | -0.0656** |  | -0.0097 | 0.0246 | 0.0484 | -0.0292* |
|  | (0.0266) | (0.0394) | (0.0653) | (0.0317) |  | (0.0129) | (0.0399) | (0.0420) | (0.0165) |
| White collar | -0.0066 | 0.0428* | 0.0112 | -0.0053 |  | -0.0178 | 0.0170 | 0.0382 | 0.0200 |
|  | (0.0199) | (0.0243) | (0.0375) | (0.0321) |  | (0.0144) | (0.0409) | (0.0463) | (0.0163) |
| Married | -0.0325 | -0.0920 | -0.0958 | 0.0514 |  | -0.0020 | 0.0222 | 0.0238 | -0.0432 |
|  | (0.0237) | (0.0644) | (0.0608) | (0.0373) |  | (0.0173) | (0.0419) | (0.0982) | (0.0332) |
| Children up to 6 years old | 0.0600 | 0.0000 | -0.1540 | 0.0539 |  | -0.0656** | 0.0000 | -0.2416* | 0.1017 |
|  | (0.1097) | (.) | (0.0984) | (0.1174) |  | (0.0300) | (.) | (0.1380) | (0.0678) |
| Children up to 18 years old | -0.0083 | 0.2664*** | -0.0378 | -0.0751* |  | -0.0128 | 0.0346 | 0.0150 | 0.0147 |
|  | (0.0295) | (0.0956) | (0.0418) | (0.0388) |  | (0.0272) | (0.0334) | (0.0363) | (0.0394) |
| Income, normalized | -0.0088 | 0.0262 | -0.0704** | -0.0009 |  | -0.0088 | -0.0020 | -0.0193 | -0.0012 |
|  | (0.0105) | (0.0323) | (0.0306) | (0.0179) |  | (0.0056) | (0.0284) | (0.0210) | (0.0112) |
| Constant | 0.0553* | 0.0277 | 0.0567 | 0.1884*** |  | 0.1358*** | 0.2912*** | 0.0307 | 0.2117*** |
|  | (0.0334) | (0.0845) | (0.0952) | (0.0621) |  | (0.0327) | (0.0959) | (0.0993) | (0.0614) |
| No. of workers | 804 | 91 | 82 | 550 |  | 804 | 91 | 82 | 550 |

***Notes***: Significance levels: * 10%, ** 5%, *** 1%. Standard errors in parentheses are clustered at the person level and calculated using sample weights. Samples: Treatment groups where the labour market state before entering temporary full-time employment is available from the survey. Original labour market state: (1) Full-time permanent employment, (2) part-time permanent employment, (3) part-time temporary employment, and (4) unemployment. All regressions contain indicator variable controls for age and quarter. Pre-treatment is an indicator variable for the last quarter before entering the first treatment. The indicator for temporary employment for, e.g., Quarter 1-5 takes the value 1 for each of the 5 quarters of temporary employment and 0 otherwise. The reference person is a blue-collar worker with middle-level education of age 35-39 in the first quarter of 2012.
