# Supplementary material for: Involuntary temporary work and mental health medications: A longitudinal study in Denmark
Source: PLOS Glob Public Health. 2023 Nov 30;3(11):e0002634. doi: 10.1371/journal.pgph.0002634 (PMC10688703; doi:10.1371/journal.pgph.0002634)
Supplement: S4 Table — (DOCX) [file pgph.0002634.s004.docx]

**S4 Table.** Involuntary temporary full-time employment and mental health compared to control group, quarterly observations, 2006-2018. Dependent variable: indicator for drug prescription each quarter.

|  |  | Women | Men |
| --- | --- | --- | --- |
| Treatment group | | 0.0037 | 0.0010 |
|  |  | (0.0056) | (0.0051) |
| Pre-treatment, 1 quarter | | -0.0138 | 0.0050 |
|  |  | (0.0086) | (0.0094) |
| Temporary employment | | |  |
|  | Quarter 1 | -0.0115* | 0.0035 |
|  |  | (0.0069) | (0.0063) |
|  | Quarter 1-2 | -0.0037 | -0.0129 |
|  |  | (0.0130) | (0.0122) |
|  | Quarter 1-4 | -0.0342 | -0.0157 |
|  |  | (0.0255) | (0.0276) |
|  | Quarter 1-5 | -0.0040 | 0.0257 |
|  |  | (0.0324) | (0.0285) |
|  | Quarter 1-6 | 0.1360* | -0.0072 |
|  |  | (0.0711) | (0.0118) |
| Post treatment | | -0.0062 | 0.0040 |
|  |  | (0.0070) | (0.0061) |
| Post treatment, quarter 1-6 | | 0.0926** | -0.0408 |
|  |  | (0.0441) | (0.1118) |
| Education level | |  |  |
|  | Low | 0.0030 | 0.0208 |
|  |  | (0.0178) | (0.0147) |
|  | High | -0.0223** | 0.0062 |
|  |  | (0.0090) | (0.0097) |
| White collar | | 0.0003 | -0.0006 |
|  |  | (0.0075) | (0.0080) |
| Married | | -0.0108 | 0.0044 |
|  |  | (0.0081) | (0.0097) |
| Children up to 6 years old | | -0.0097 | -0.0106 |
|  |  | (0.0142) | (0.0127) |
| Children up to 18 years old | | 0.0030 | -0.0196* |
|  |  | (0.0117) | (0.0105) |
| Income, normalized | | -0.0084** | -0.0037** |
|  |  | (0.0042) | (0.0019) |
| Constant | | 0.1648*** | 0.1163*** |
|  |  | (0.0142) | (0.0132) |
| No. of persons | | 5,345 | 3,805 |

*Notes*: Significance levels: * 10%, ** 5%, *** 1%. Standard errors in parentheses are clustered at the person level and calculated using sample weights. The control group is formed from observations from workers that enter temporary employment at a later point in time and by assigning a “placebo” transition to temporary employment. The table displays the coefficients from interaction terms between the treatment sample indicator and the treatment indicators in fixed effects regressions. Pre-treatment is an indicator variable for the last quarter before entering the first treatment. The indicator for temporary employment for, e.g., Quarter 1-6 takes the value 1 for each of the 6 quarters of temporary employment and 0 otherwise. The data encompasses persons who had full-time employment in at least one of the LFS surveys in 2006-2018 and include mental health indicators of the participants eight quarters before the first survey and eight quarters after the last survey.
