# Supplementary material for: Involuntary temporary work and mental health medications: A longitudinal study in Denmark
Source: PLOS Glob Public Health. 2023 Nov 30;3(11):e0002634. doi: 10.1371/journal.pgph.0002634 (PMC10688703; doi:10.1371/journal.pgph.0002634)
Supplement: Attachment — Submitted filename: Review_PLos Global Health.docx [file pgph.0002634.s005.docx]

Review: PGPH-D-23-00532

Very interesting and innovative research topic.

Some points need to be tackled to improve the manuscript:

**Title:**

Adjust the title to make it more specific about your research topic and the research methods.

My suggestion:

Involuntary temporary work and mental health medications: a longitudinal study in Denmark

**Abstract;**

Make somewhere already clear that you only selected full-time workers.

**Throughout manuscript (and abstract):**

Pay attention to your wording; this is an article about “Involuntary” full-time temporary employment, yet throughout the description of your results and in the discussion, the word “involuntary” is often omitted making the manuscript confusing and hard to follow (as the reader is believed to think that the results are about temporary employment (independent of being involuntary). Add the word “involuntary” to the text where necessary.

The authors highlight too much that their study is on mental health. Their study is only on the use of medication for certain mental health problem. Many people who have (common) mental health problems do not use medication. The authors should be more transparent about how specific their study is and use the wording “the use of medication for mental health problems” instead of mental health as much as possible.

**Introduction**:

P 2: 3 paragraph: “this is the first study to do a separate analysis” – what do you mean by “separate analysis”. Lots of studies are done about the health effects of temporary employment.

More information (e.g. previous research) should also be given about what is already know about medication for mental health problems and temporary employment.

**Methodology**

Add more information about the data, what are the years that these data were gathered?

More information is also needed about who is selected into the group that are analyzed: there age-range.

A visualization of the data and used gathered would be a useful tool to understand the data better. The authors report on a “pause” in the collecting of the survey. This will also become more clear how this affected the data and results, with a visualization.

Be more specific about what a quarter means (is this 3 month?) – could also be helpful in the tables.

Can you control your results for income and family composition, as these are an important confounder between employment and mental health. The authors should think about more variable which could be added as controls.

Describe also what methodology is used for the descriptive tables.

Describe whether you used weights, how you handled missing data, whether some variables had high missing data.

In the end you do an analysis with part-time employed; this should already be explained in the methodology.

**Results**

This will be an article which will be interesting to many occupational sociologists. The use of the medical words “Treatment group” and “pre-treatment”, “post treatment”, etc. is difficult to follow when not being from a medical background. These words should be replaced by the “labels” which they stand for “such as, permanent, and involuntary temporary employment. This will make the interpretation of the results, tables and figure much more clarity.

**Tables**

Avoid to use of the words “quarter” it is very difficult to understand.

**Figure & Tables:**

What is pre-treatment? Unemployed?

Post-treatment? Not being involuntary temporary employed anymore?

**Discussion**

Be specific when you mention “shorter periods” – how many months are that?

P 13, first sentence above: is that compared to fulltime permanent workers? Please be specific.

The authors mention that “since the mid-1990 “non-standard” jobs increased. However, within the occupational sociology, scholars usually mention the 1970’s as the start of more flexible contracts.

See Benach et al 2014 Precarious Employment: Understanding an Emerging Social Determinant of Health. Annual Review of Public Health

P14 Last sentence of the discussion: “When some women choose to enter temporary employment” is this really an issue in your study when you only selected “involuntary” temporary workers?

**Conclusion:**

Second sentence: “workers on temporary contract has worse mental health before entering “temporary employment ….

This sentence is confussing - what do you mean? What was there status then before they entered temporary employment? Unemployed?
