# Supplementary material for: Involuntary temporary work and mental health medications: A longitudinal study in Denmark
Source: PLOS Glob Public Health. 2023 Nov 30;3(11):e0002634. doi: 10.1371/journal.pgph.0002634 (PMC10688703; doi:10.1371/journal.pgph.0002634)
Supplement: Attachment — Submitted filename: PGPH-D-23-00532_review.docx [file pgph.0002634.s006.docx]

PLOS Global Public Health

Manuscript Number: PGPH-D-23-00532

Full Title: Involuntary temporary work and mental health in Denmark

The main aim of the paper is to bridge the gap in the literature with regard to the relationship between involuntary temporary employment and mental health. The authors estimate gendered effects using various estimation measures and assess the medium-term effect of being in involuntary employment. In general, the paper is well-written and conveys its main messages clearly. In the following, I comment on a number of issues that need revision or clarification.

**General comments**

1. The paper is missing a section that tackles the theoretical background and the country-specific background.

**Introduction**

2. It would be interesting to add some statistics about the involuntary temporary employment in Europe in general and Denmark in particular to show the relevance of studying this topic.

**Methods**

3. The authors excluded part-time employees and only included in their sample full-time employees. A reasoning is needed for excluding this category especially since women usually represent a large percentage of part-time workers.
4. Since the authors conduct heterogeneous analysis by gender, I expect that the models include some other control variables such as the marital status, the number of children and the age of the children.
5. The assumptions of the various models need to be highlighted and the differences in biases between the the three models. This should also be reflected in the interpretation of the results.
6. I am assuming from the below sentence in the results section that the authors restricted the sample to those who were unemployed and transitioned to temporary employment. Could this be clarified?

“The main results in the study stems from fixed effects longitudinal models, where we assess mental health during and after temporary employment relative to workers health before entering temporary employment.”

7. As a follow-up to the previous point, in the exposure or treatment variable sub-section, the authors need to add the description/operationalization of the treatment variable for each model separately.
8. Regarding the matching diff-in-diff, could the authors add more details with regard to the variables used in the matching, which algorithm is chosen, what are the balancing results?

**Results**

9. Using the term lasting effects is misleading given that the effect is only calculated for a number of quarters later. I would suggest using medium-term effect instead.
10. The following paragraph is unclear and need further explanation as to what is meant by previous level. “After temporary employment, drug use returns the previous level for most spell lengths. The exception is women with spell lengths of six quarters of temporary employment for which a separate indicator provides evidence of increased use of drugs.”
11. It would be interesting to dig deeper in the results for men. They show a different trend compared to women with regard to sign and magnitude. Despite being insignificant, it is still useful to analyze these results, following the recommendations by Bernardi et al. (2017).

**Discussion**

12. Could you please specify what is the treatment variable used in the paper by Bardasi and Francesconi (10)? This will be useful in the discussion section to see how far the two definitions are.
13. Only in the discussion that we read about the fact that the temporary employment could have started before the first quarter when the individual is first captured in the data. In light of this, the authors should be careful with the use of the terms short and long-term results of temporary employment and entering temporary employment. This issue deserves thorough discussion in the methods section.

**References**

Bernardi, F., Chakhaia, L., & Leopold, L. (2017). ‘Sing me a song with social significance’: the (mis) use of statistical significance testing in European sociological research. European Sociological Review, 33(1), 1-15.
