# Supplementary material for: Involuntary temporary work and mental health medications: A longitudinal study in Denmark
Source: PLOS Glob Public Health. 2023 Nov 30;3(11):e0002634. doi: 10.1371/journal.pgph.0002634 (PMC10688703; doi:10.1371/journal.pgph.0002634)
Supplement: Attachment — Submitted filename: Resonse_referees (v6).docx [file pgph.0002634.s007.docx]

**REVIEWER 1**

1. **Title: Adjust the title to make it more specific about your research topic and the research methods. My suggestion: Involuntary temporary work and mental health medications: a longitudinal study in Denmark.**

We thank the reviewer for this highly relevant comments on the importance of being specific about the type of mental health problems that we investigate in the paper (please also see our response to comment #4). As a response, we have changed the title of the paper to “Involuntary temporary work and mental health medications: A longitudinal study in Denmark”.

2. **Abstract: Make somewhere already clear that you only selected full-time workers.**

Throughout our revised abstract, we now use the term “involuntary full-time temporary employment”.

3. **Throughout manuscript (and abstract). Pay attention to your wording. This is an article about “Involuntary” full-time temporary employment, yet throughout the description of your results and in the discussion, the word “involuntary” is often omitted making the manuscript confusing and hard to follow (as the reader is believed to think that the results are about temporary employment (independent of being involuntary). Add the word “involuntary” to the text where necessary.**

In response to this comment, we have added the word “involuntary” to the text in the revised manuscript where necessary (highlighted with yellow in the manuscript).

4. **The authors highlight too much that their study is on mental health. Their study is only on the use of medication for certain mental health problem. Many people who have (common) mental health problems do not use medication. The authors should be more transparent about how specific their study is and use the wording “the use of medication for mental health problems” instead of mental health as much as possible.**

This point is an important one that needs elaboration. In addition to the changes we made in our abstract as a response to comment #1, we have changed the title of the subsection on page 8 of the revision that present our operationalization of health problems into “Medication for certain mental health problems.” Moreover, throughout our revised manuscript, we have changed the terms “outcome variable” and “mental health problems” into “medication for mental health problems” (see for example pages 9 and 11). In addition, in the revised manuscript, we have added the following sentences on page 8:

While a number of studies have used medication as a measure of mental health problems, we are aware that many people with similar health problems (for various reasons) do not use medication. Our analyses should therefore be seen as relatively conservative estimates, as the number of people with mental health problems due to involuntary temporary employment may be significantly higher.

5. **Introduction: P 2: 3 paragraph: “this is the first study to do a separate analysis” – what do you mean by “separate analysis”. Lots of studies are done about the health effects of temporary employment.**

We have rewritten the sentence. The revised sentence on page 2 now reads as follows: “First, our study is the first to conduct analyses of the consequences of *involuntary* temporary employment on mental health.”

6. **More information (e.g. previous research) should also be given about what is already know about medication for mental health problems and temporary employment.**

In response to this comment, pages 4–6 in the revised manuscript now include a section about previous research analysing the link between temporary employment and medication for mental health problems.

7. **Methodology. Add more information about the data, what are the years that these data were gathered?**

We now provide more details about the survey data collection. For example, page 7 in the revised manuscript now reads:

We use data from the European Union Labour Force Survey (LFS), a longitudinal or panel survey that interviews participants up to four times. The second survey takes place 3 months after the first survey; third and fourth surveys take place after a 6 months break. In our analysis, we only use data from the rounds collected between 2006 and 2018 and limit the analysis to respondents aged 20-64. Figure 1 illustrates how many respondents that participated in each wave of the survey.

[FIGURE 1 AROUND HERE]

As shown in Figure 1, the majority of the respondents (94 pct.) participated in at least two rounds. After the two quarter pause (6 month), 78 pct. of the respondents participated in the third survey. More than half of the respondents (59 pct.) also participated in the fourth round of the survey.

8. **More information is also needed about who is selected into the group that are analyzed: there age-range.**

Please see our response to comment #7. As stated on page 7, the respondents are between ages 20–64. We have added the following sentence to the description of the group that are analysed:

The lower age of temporary workers is reflected in a lower marriage rate and lower shares of workers with children.

9. **A visualization of the data and used gathered would be a useful tool to understand the data better. The authors report on a “pause” in the collecting of the survey. This will also become more clear how this affected the data and results, with a visualization.**

Please see our response to comment #7.

10. **Be more specific about what a quarter means (is this 3 month?) – could also be helpful in the tables.**

We now explain that a quarter is three months. Please see comment #7.

11. **Can you control your results for income and family composition, as these are an important confounder between employment and mental health? The authors should think about more variable which could be added as controls.**

We thank the reviewer for reminding us of the importance of using the relevant background variables. In our administrative data, we have the possibility to code time variables that measure income and family composition. As a response, page 9 in our revised manuscript now reads:

Inspired by previous research (28), our regression models include control variables for the workers’ education, occupation and income. We draw on the International Standard Classification of Education to classify the workers’ education into three categories: “high” (university or college), “middle” (vocational or high school), and “low” (below high school). The occupational categories are white-collar workers (main categories 0–4 in the International Standard Classification of Occupations, ISCO), and blue-collar workers (main ISCO categories 5-9). Information about income come from tax registers. From these registers, we apply a variable measuring the annual total income that includes earnings, savings and other capital assets. We also include demographic household variables about family composition (i.e., marital status, an indicator for children below age 6 living in the household, and an indicator for children below age 18 living in the household).

We have estimated all models again with the new background variables included. All tables and associated body text have been updated. The new background variables have however no substantial impact on the overall conclusions of our analyses. For example, for both women and men, the coefficients for our key variable for involuntary temporary employment only change to the fourth decimal place.

12. **Describe also what methodology is used for the descriptive tables.**

On page 11, we now explain that Table 1 presents summary statistics using means and the standard deviation.

13. **Describe whether you used weights, how you handled missing data, whether some variables had high missing data.**

Thanks to this comment, we now provide more details about how we handled missing data. Pages 10-11 in the revised manuscript under the section “Methodology and empirical strategy”, now reads as follows:

We use sample weights for calculating all the statistics that are reported in the paper. The sample weights of the LFS takes both stratification and attrition into account. For each respondent, we apply the average of the sample weights in the surveys. We handle missing values as follows: Respondents without educational information are omitted from the analyses, corresponding to 2.3 pct. of the observations; we assign workers zero children if there is no information about children in the data; workers without marriage information are treated as singles; six workers without occupational information are categorized as blue-collar workers on the basis of their educational information.

14. **In the end you do an analysis with part-time employed; this should already be explained in the methodology.**

The revised section “Methodology and empirical strategy” on page 10 now ends with the following paragraph:

While our main focus is on involuntary full-time temporary employment, we also include workers with part-time employment in subsection of the analysis entitled “Involuntary temporary employment and previous labour force states”. In this part of the analysis, we assess the role of previous labor market states before the worker enters involuntary full-time temporary employment, which might include part-time employment.

15. **Results. This will be an article which will be interesting to many occupational sociologists. The use of the medical words “Treatment group” and “pre-treatment”, “post treatment”, etc. is difficult to follow when not being from a medical background. These words should be replaced by the “labels” which they stand for “such as, permanent, and involuntary temporary employment. This will make the interpretation of the results, tables and figure much more clarity.**

We thank the reviewer for this suggestion, which will certainly make the manuscript easier to read. In the revised manuscript, we have followed the reviewer's suggestion and changed all references in both the main text and in tables/figures from e.g. "treatment group" and "comparison group" into "involuntary temporary employment" or "permanent full time employment"

16. **Tables. Avoid to use of the words “quarter” it is very difficult to understand.**

In the revised version, we have changed all tables from referring to quarters to months. The body text of the tables has also been corrected.

17. **Figure & Tables. What is pre-treatment? Unemployed? Post-treatment? Not being involuntary temporary employed anymore?**

In the revised manuscript, we have changed all tables and figures so that they no longer refer to these terms, but instead refer to, for example, the group of involuntary temporary workers or the group of workers in full employment.

18. **Discussion. Be specific when you mention “shorter periods” – how many months are that?**

As a response to this comment, we have strived in our revision of the manuscript to be even more precise. We therefore no longer use phrases such as "shorter periods" or "longer periods", but instead indicate time in months. For example, page 9 in the revised manuscript now reads:

Fourth, after leaving involuntary temporary employment, we found no permanent effects of shorter spells of temporary employment (less than 12 months) on prescription drugs for mental health problems.

19. **P 13, first sentence above: is that compared to fulltime permanent workers? Please be specific.**

Thanks to this comment, throughout the manuscript, we now mention the reference group every time we discus effects.

20. **The authors mention that “since the mid-1990 “non-standard” jobs increased. However, within the occupational sociology, scholars usually mention the 1970’s as the start of more flexible contracts.**

As a response to this comment, throughout the revised manuscript, we have now changed our description of the period to “since the 1970s”.

21. **See Benach et al 2014 Precarious Employment: Understanding an Emerging Social Determinant of Health. Annual Review of Public Health**

We thank for reviewer for this highly useful references that is included in the revised manuscript.

22. **P. 14 Last sentence of the discussion: “When some women choose to enter temporary employment” is this really an issue in your study when you only selected “involuntary” temporary workers?**

As a response to this comment we have removed the word “choose” from the sentence. The sentence on pages 19–20 to read as follows:

Another potential reason for this negative coefficient is self-selection, which is the case when some women (due to different life circumstances such as the rules for receiving social benefits) enter involuntary temporary employment when they experience improved mental health.

23. **Conclusion: Second sentence: “workers on temporary contract has worse mental health before entering “temporary employment …. This sentence is confusing - what do you mean? What was there status then before they entered temporary employment? Unemployed?**

In response to this comment, we rewritten the sentences. Page 20 now reads as follows:

First, compared to workers on permanent employment contracts, workers on temporary contracts had worse mental health before transitioning into involuntary temporary employment.

**REVIEWER 2**

1. **The paper is missing a section that tackles the theoretical background and the country-specific background.**

We thank this reviewer for this important comment. In the revised manuscript on pages 3–6, we now include a new section entitled ‘Background’. In this section, we present the theoretical background of our analysis and give a presentation of the Danish setting.

2. **It would be interesting to add some statistics about the involuntary temporary employment in Europe in general and Denmark in particular to show the relevance of studying this topic.**

Please see our response to comment #1. In the new background section, we have added some statistics about the rise in temporary employment in Europe and Denmark.

3. **The authors excluded part-time employees and only included in their sample full-time employees. A reasoning is needed for excluding this category especially since women usually represent a large percentage of part-time workers.**

In this paper, we have chosen to focus solely on involuntary temporary employment. We strongly agree with the reviewer that more women than men have short-term employment and that it would therefore be interesting to investigate gender differences for this type of employment. However, we believe that such an analysis would be outside the focus of this paper, where we have chosen to focus solely on the issue of involuntary temporary (full-time) employment.

4. **Since the authors conduct heterogeneous analysis by gender, I expect that the models include some other control variables such as the marital status, the number of children and the age of the children.**

We strongly agree with the reviewer and have therefore included several control variables in our analyses (including marriage and number of children). See also our response to reviewer 1's comment #11.

5. **The assumptions of the various models need to be highlighted and the differences in biases between the three models. This should also be reflected in the interpretation of the results.**

In response to this comment, the section on results after the presentation of the OLS (Model 1) and fixed effects (Model 2) estimations on page 14, we now include the following paragraph:

The OLS estimates in Model 1 does not take unobserved differences between workers into account. For example, workers who enter involuntary full-time temporary employment may have certain personality traits that interacts with the both the outcome variable (mental health prescription drugs) and the treatment variable (involuntary temporary employment). By applying fixed effects estimation, model 2 enable us to take time-invariant unobserved differences between workers (such as personality traits) into account. The estimates in Model 1 of the effect of involuntary temporary employment on mental health will thus be downward biased if workers inclined to use prescription drugs tend to avoid entering involuntary temporary employment. Such a bias might be the reason why the coefficients on involuntary temporary employment in Model 2 is larger than the ones in Mode1 for lengths of duration beyond 3 months.

In the section about control groups on page 17 in the revised manuscript, we now include an explanation about the “difference-in-difference” matching procedure:

As a supplement to the fixed effects models, we therefore assess the development of the mental health of temporary workers relative to a control group by applying “difference-in-difference matching” (16). This procedure has the potential of adjusting for the bias in the fixed effects estimates that arises if the use of prescription drugs among temporary employed workers changes over time for other (unobserved) reasons than changes in employment contracts. We apply workers who enter temporarily employment in later point of time as control groups (see (14) and (15) for details on this recent procedure). The results from matching models that include control groups are shown in Table A.4 in the Appendix. Results from these matching models deviate only marginally from the results of the regression models without control groups shown in Table 2.

6. **I am assuming from the below sentence in the results section that the authors restricted the sample to those who were unemployed and transitioned to temporary employment. Could this be clarified? “The main results in the study stems from fixed effects longitudinal models, where we assess mental health during and after temporary employment relative to workers health before entering temporary employment.”**

In our analysis, we include all workers who entered involuntary temporary employment. We thank the reviewer for making us aware that our wording could be misunderstood. We have therefore rewritten the sentence. In the revised manuscript, page 17 now read as follows:

The main results in the study stems from fixed effects longitudinal models, where we assess the use of prescription drugs for mental health during and after involuntary temporary employment relative to workers’ health before entering temporary employment. In these analyses, we do not distinguish whether the workers were unemployed or came from other types of employment before entering involuntary full-time temporary employment.

7. **As a follow-up to the previous point, in the exposure or treatment variable sub-section, the authors need to add the description/operationalization of the treatment variable for each model separately.**

Again, we thank the reviewer for making us aware of the need to be clearer in our wording. On page 8 in the revised manuscript, we now include the following sentence that explain how we divide our treatment variable (i.e., involuntary temporary employment) into different lengths of employment:

In our analyses, we include variables that measure different length of involuntary temporary employment varying from 3 to 18 months of employment.

8. **Regarding the matching diff-in-diff, could the authors add more details with regard to the variables used in the matching, which algorithm is chosen, what are the balancing results?**

In the section on control groups on page 17, we have inserted a revised explanation of the “difference-in-difference” matching procedure:

This procedure has the potential of adjusting for the bias in the fixed effects estimates that arises if the use of prescription drugs among temporary employed workers changes over time for other (unobserved) reasons than changes in employment contracts. We apply workers who enter temporarily employment in later point of time as control groups (see (14) and (15) for details on this recent procedure).

A main idea in using observations from the intervention group at later points of time as controls is that the intervention and the control group is more or less balanced from the outset and that formal tests of balancing thus passes per construction. In line with (14) and (15), the revised manuscript does thus not contain formal balancing tests.

9. **Using the term lasting effects is misleading given that the effect is only calculated for a number of quarters later. I would suggest using medium-term effect instead.**

As a response to this comment (and reviewer 1 comment #18), we have we have changed all time references to be based on months instead. For example, in the revised manuscript on page 15, we now write:

Furthermore, involuntary temporary employment does not appear to have effects on mental health 18 months after

And page 19, we now write the following:

Third, for women we found substantial adverse effects of involuntary temporary employment on mental health for temporary contracts between 15 and 18 months

In addition, we have also changed the time references in the abstract.

10. **The following paragraph is unclear and need further explanation as to what is meant by previous level. “After temporary employment, drug use returns the previous level for most spell lengths. The exception is women with spell lengths of six quarters of temporary employment for which a separate indicator provides evidence of increased use of drugs.”**

Thanks to his comment we have rewritten the paragraph. On page 14 in the revised manuscript, we now write:

After involuntary temporary employment, the level of drug use return to the previous level the worker had 4–24 months before they are observed entered involuntary temporary employment. The exception is women with 15–18 months of involuntary temporary employment for which a separate indicator provides evidence of increased use of drugs 24 months after being observed in involuntary full-time temporary employment. Women with 15–18 observed months of involuntary temporary employment appear to experience decreased mental health both during and after involuntary temporary employment.

11. **It would be interesting to dig deeper in the results for men. They show a different trend compared to women with regard to sign and magnitude. Despite being insignificant, it is still useful to analyze these results, following the recommendations by Bernardi et al. (2017).**

We very much agree that it would be interesting to do more in-depth analyses of the men, but unfortunately, this is beyond the focus of this article. We also agree with the points made in the excellent paper by Bernardi et al. (2017). Inspired by this paper, we now write in the conclusion that future studies could benefit from further investigations into the differences between men and women in involuntary temporary employment. Thus on page 20 in the revised manuscript, we write as follows:

In addition, as we find different patterns for men compared to women with regard to sign and magnitude of the effects, future research should investigate gender differences in the effects of involuntary temporary employment.

12. **Could you please specify what is the treatment variable used in the paper by Bardasi and Francesconi (10)? This will be useful in the discussion section to see how far the two definitions are.**

As a response to this comment, we have rewritten our discussion of our results in relation to the findings reported in the paper by Bardasi and Francesconi**.** Page 17 in the revised manuscript now reads:

We are however not the first to emphasize the importance of separate analysis of involuntary temporary employment. For example, Bardasi and Francesconi (10) use survey data to find that workers with seasonal or causal work, or work done under contract or a fixed period of time in the UK did not have long-lasting detrimental mental health effects for either female or male workers. They note that due to data limitations they cannot distinguish between involuntary and voluntary temporary employment and that health effects may even be positive.

13. **Only in the discussion that we read about the fact that the temporary employment could have started before the first quarter when the individual is first captured in the data. In light of this, the authors should be careful with the use of the terms short and long-term results of temporary employment and entering temporary employment. This issue deserves thorough discussion in the methods section.**

We no longer use the terms short and long employment. In the revised manuscript, we only use months to describe the duration of involuntary temporary employment.
