# Supplementary material for: Involuntary temporary work and mental health medications: A longitudinal study in Denmark
Source: PLOS Glob Public Health. 2023 Nov 30;3(11):e0002634. doi: 10.1371/journal.pgph.0002634 (PMC10688703; doi:10.1371/journal.pgph.0002634)
Supplement: Attachment — Submitted filename: Response to reviewers.docx [file pgph.0002634.s008.docx]

**EDITOR COMMENTS**

**Before, accepting your manuscript to PLOS Global Public Health, I would like you to**

**address the following:**

**1) Reviewer's 2 comment number 13 (Only in the discussion that we read about the fact that the temporary employed could have started before the first quarter, when the individual is first captured in the data. In light of this the authors should be careful with the use of the terms short and long-term results of temporary employment and entering temporary employment. This issues deserves thorough discussion in the methods section).**

**I'm not satisfied with the adjustments made by the authors (only using months to describe the duration). I would like to see, just like Reviewer 2, a discussion about this issues in the methods**

**section already and how this would affect your results.**

**Moreover, in the sensitivity analysis The authors mention, we confine the analysis to those workers where the labour market status is observed before entering "involuntary temporary employment" (ITE). Why is it possible that the authors can observe the labour market status before entering ITE, and at the same time the temporary employed could have started before the first quarter? Can you not also exclude those who started ITE before the first quarter then?**

Thanks for your comments. We have amended the manuscript such that it is now hopefully more clear what kind of data we use for the different analyses in the paper. We have followed the advice and included extra explanations in the methods section. This includes a new version of Figure 1. We made the previous version of figure 1 in an attempt to make the composition of the data more transparent. This was obviously not successful and we have thus made a new attempt to describe the quarterly data collection of the European Labour Force Survey. For the same reason, we also return to characterize the timing to quarters instead of months. As a result, pages 7–8 in the revised manuscript now read as follows:

The data come from the European Union Labour Force Survey (LFS), which is a quarterly comparative panel survey that interviews a sample of people in the European countries about their labour market participation. The respondents are interviewed up to four times. The second interview takes place one quarter after the first survey, while the third and fourth interviews take place after a two-quarter pause. In our analysis, we use interview rounds collected between 2006 and 2018 and limit our sample to respondents between ages 20 and 64.

Figure 1 illustrates the data collection in LFS for the first quarter of 2006 to the last quarter of 2008. The first group, labelled group A, is interviewed in the first and the second quarter of 2006. After a break during the third and fourth quarter of 2006, the group is interviewed again in the first and the second quarter of 2007.

**Figure 1**. Illustration of the data collection process for the Labour Force Survey for eight rounds of interviews between 2006 and 2008, where grey boxes indicate that the respondent group is invited for an interview.

Group B starts the first interview in the second quarter of 2006 and is interviewed for the last time in the third quarter of 2007. The LFS continues to add new respondents in each new quarter, not only for the groups C to H that are included in Figure 1 but up to 2018, which is the last year of our data.

In our main analysis, we include all respondents who have answered at least once that they are in ITE. If a respondent states they are in ITE in the first quarter of the LFS, we do not have information about their previous labour market status. We complement the main analysis with an analysis of how the consequences of ITE might differ depending on the worker’s previous labour market status. This complementary analysis demands that a labour market state is observed before the first quarter in which respondent answers that she is in ITE. As a result, the complementary analysis only includes respondents who have participated a maximum of three times in the LFS.

**2) Reviewer's 3 comment number 3 (The authors excluded part-time employees and only included in their sample full-time employees. A reasoning is needed for excluding this category especially since women usually represent a large percentage of part-time workers).**

**I believe the authors are not answering reviewer's 2 comment correctly. Reviewer 2 is talking about part-time work and the authors are answering about temporary or short-term employment, this is not the same. Could you please mention in the methods section already what the reasoning was to exclude part-time workers and how this would affect your results (since women usually are part-time workers).**

Thanks for this comment. We now mention in the methods section why we exclude part-time workers. On page 8 in the revised paper, the text reads now reads as follows:

We confine the analysis to workers in full-time involuntary temporary employment only. Our reason for not including part-time temporary workers is that full-time workers might exhibit higher levels of organizational commitment and job satisfaction compared to part-time employees. As a result, mental health consequences of temporary employment might also differ for full-time and part-time workers. Inclusion of part-time workers in the analysis would thus make our results a mixture between the responses of the two groups of workers.

We hope that we now provide a sufficient explanation for the exclusion of part-time workers. In the revised paper, we now also analyse a group of workers that is homogeneous with respect to working time (part-time/full-time). Heterogeneous groups included in the same analysis might result in heterogeneous responses.

**3) Figure 1 needs some more work. It is still very difficult to understand the way the data is gathered. In the manuscript it is talked about 4 waves and in the figure I see 6 rounds? Could you also add the years (2006, 2018, etc...) to your figure, name your x and y-axis, as a reader I want to be able to understand the "months" which the article mentions so much.**

As described above, we have removed Figure 1 in the previous version of the paper and have replaced it with a new figure that illustrates the data collection process. We hope that it is now easier to understand the way the data is gathered in the European Labour Force Survey. Furthermore, we characterize the timing by quarters instead of months.

**4) Sensitivity analyses (Involuntary temporary employment and previous labour force status). Please do not call part-time employment or voluntary temporary employment "precarious employment". This is not at all in line with consensus about precarious employment. Part-time employment can be "protected" employment because of good employment conditions, the same holds for temporary employment. Precarious employment can be considered a multidimensional construct and only measuring it with 1 is not the best way to do it. Group 2 talking part-time workers and voluntary temporary workers together is not the best idea? split them up?**

Thanks for the comments and suggestions. We have amended the manuscript to comply with your suggestions. The revised manuscript no longer characterises part-time employment and voluntary temporary employment as “precarious employment”. This concept is now mentioned in the beginning of the paper in the sections on background and literature but not in the rest of paper. The tables (Tables A.2 and A.3) for the section on “Involuntary temporary employment and previous labour force status” now contain separate analyses of the full-time involuntary temporary workers who came from part-time permanent jobs and those who come from part-time temporary jobs.

**5) Please read your article carefully for typo's.**

A native English-speaking editor has proofread the revised paper.

**6) Table 2, Model 2 and 4, for the sake of clarity please write in the notes the comparison group of these models.**

In the revised paper, the notes now contain a description of the comparison implied by the models.
